# Challenges in Managing Paediatric Crohn’s Disease With Crohn’s Disease Exclusion Diet (CDED): The First Single-Center Study in the United Arab Emirates

**DOI:** 10.7759/cureus.43970

**Published:** 2023-08-23

**Authors:** Ali Alsarhan, Rehab Aljasmi, Natacha Ajaka, Balaji Krishnamurthy, Ajmal Kader, Masooma Aljasmi, Noor Nahdi, Ehsan Malik, Buthaina Murbati, Eiman Aljabri, Christos Tzivinikos

**Affiliations:** 1 Pediatric Gastroenterology, Al Jalila Children's Speciality Hospital, Dubai, ARE; 2 Medical Affairs, Al Jalila Children's Speciality Hospital, Dubai, ARE; 3 Nutrition, Al Jalila Children's Speciality Hospital, Dubai, ARE; 4 Pediatric Gastronetrology, Al Jalila Children's Speciality Hospital, Dubai, ARE

**Keywords:** nutrition, arab, united arab emirate, crohn's disease exclusions diet, crohn’s disease (cd)

## Abstract

Background: Inflammatory bowel disease has been increasing significantly. For that, many modalities of treatment have shown promising results including a special diet. In our study, we looked at Crohn’s disease dietary management for induction and subsequently maintenance of treatment. The research question was how feasible was applying this approach and what difficulties the patients and their parents faced.

Methodology: We reviewed the electronic medical system. We had 32 paediatric patients who were diagnosed with Crohn's disease and used the special diet plan (Crohn’s disease exclusion diet or exclusion enteral nutrition) to induce remission or maintenance. Then, we used a questionnaire to identify the difficulties they faced while using the special diet.

Results and discussion: We have found that the cohort had used the special diet for a variable duration. The majority of patients opted to start with Crohn's disease exclusion diet. The clinical response was inconsistent due to poor compliance. Only 57 % of the patients were able to bear with the dietary plan beyond 12 weeks. Patients reported the following factors which caused non-compliance: intolerance/difficulty to follow (40%), cost (19%), poor clinical response (19%), and others.

Conclusions: In managing Crohn's disease, a multidisciplinary approach, including dietary interventions, is crucial. However, adherence to specialized diets poses several challenges identified in this study based on patient feedback. Addressing barriers and prioritizing dietitians' role is essential for improved patient outcomes in inflammatory bowel disease management.

## Introduction

Inflammatory bowel disease (IBD) is a chronic autoimmune condition that affects the digestive tract. It is characterized by inflammation and ulceration in the gastrointestinal tract, which can lead to symptoms such as abdominal pain, diarrhea, and weight loss [1]. The initial description of IBD can be traced back to the early 20th century when Crohn et al. published a landmark paper describing a new disease that they called "regional ileitis" (Crohn's disease) in 1932 [2]. Later, in 1955, Melrose described the epidemiology of ulcerative colitis disease entity [2]. Since then, there has been a significant increase in our understanding of IBD, but the exact cause of the disease is still unknown.

According to a study published in the Gastroenterology Journal, the incidence and prevalence of Crohn's disease (CD) in the paediatric population have been increasing over the past few decades. The study reports that the current range of CD incidence in children varies from 0.3 to 15.3 cases per 100,000 children per year in the middle east. Similarly, the prevalence of CD among children is estimated to be around nine per 100,000 children [3]. Another study published in the American Journal of Gastroenterology reported that the prevalence of CD in children and adolescents in the United States is approximately 48 per 100,000 individuals [4]. One of the most convincing environmental factors contributing to the increased incidence of Crohn's disease is the adoption of a Western diet characterized by the consumption of processed and ultra-processed foods [5]. The recent dietary guidance provided by the International Organization for the Study of Inflammatory Bowel Diseases advises individuals to reduce their intake of saturated and trans fats, emulsifiers, artificial sweeteners, maltodextrins, and carrageenans. These substances are frequently found in processed foods [6].

As the number of patients with CD continues to rise, healthcare providers are under increasing pressure to implement more advanced and effective treatment modalities with a better safety profile. One such modality is the use of a novel dietary treatment approach called Crohn’s disease exclusion diet (CDED), which encompasses structured dietary modifications to manage CD [7]. CDED has shown efficacy in paediatric and adult randomised control trials, demonstrating improved disease outcomes in adults and children and reduced need for medication in some patients [7]. Recent guidelines for nutrition in inflammatory bowel disease recommend that CDED plus partial enteral nutrition (EN) should be considered an alternative to exclusive EN in paediatric patients with mild to moderate CD to achieve remission [8].

CDED involves removing specific dietary components that are known to trigger inflammation and exacerbate symptoms, and replacing them with anti-inflammatory foods in addition to special milk formula [9]. The goal of CDED is to reduce inflammation and promote healing in the gastrointestinal tract while optimizing the effectiveness of biological therapy. The mechanism of the effect of CDED is not fully understood, but it is thought to involve a combination of anti-inflammatory and immunomodulatory effects [9]. On the other hand, exclusion enteral nutrition (EEN) involves the sole use of a nutritionally complete milk formula with the exclusion of any other kind of food. EEN has been found to be as effective as corticosteroids in inducing remission in children with active CD and may be more effective than corticosteroids in promoting mucosal healing [8]. The mechanism of the effect of EEN is thought to involve a combination of reduced antigen exposure, improved nutritional status, and altered gut microbiome composition [9].

It has been observed from our clinical practice that adherence to the dietary plans for managing CD can vary and fluctuate among different families and patients, which can significantly impact the success of these treatment modalities. However, there is a dearth of data on patients' and their caregivers' perspectives regarding dietary plans and the impact they have on their lives. In this study, we aim at reporting our experience at Al Jalila Children's Specialty Hospital in utilizing CDED as a therapeutic approach for the management of CD. Additionally, we seek to highlight the perspectives of patients and their families on this treatment modality. By describing our experience and considering the perspectives of patients and families, we aim to contribute to the growing body of knowledge on the use of CDED in the management of CD.

## Materials and methods

In this study, we retrospectively accessed the electronic medical records of paediatric patients with CD between 2017 and 2022 who met the inclusion criteria. Patients who reported using a specific diet plan for the induction or maintenance of remission were included in the study. Data was collected on the disease and treatment modalities of these patients. Some patients were sent a questionnaire to complete, while others preferred to respond over the phone with the investigator. The collected data was then thoroughly reviewed and analysed. The main findings of this study were highlighted and presented.

The population under investigation comprised a total of 32 patients. Both male and female patients were included, and their age (at diagnosis) distribution covered a wide range, from the very young (two months) to adolescents (17 years). The inclusion of patients from this wide age range ensures a broad representation of the paediatric population with CD, and thus increases the generalizability of the findings.

The demographic characteristics of the patients included in this study were carefully considered to ensure a representative sample of the paediatric population affected by CD. The inclusion criteria were: CD patients aged between 28 days and 18 years of age, had initiated the prescribed diet for at least one-week duration, and are readily accessible and capable of completing a comprehensive questionnaire. The exclusion criteria were: diagnosis other than CD even if dietary management was used, initiated the prescribed diet for less than one-week duration, and the patient was inaccessible and incapable of completing the comprehensive questionnaire.

## Results

In this study, a total of 32 patients were included, with a gender distribution of 56% male and 44% female. The majority of patients (62.5%) fell within a specific age range (9 and 14 years old). Among the patient population, 23 (71.8 %) were of Arab ethnicity, with 14 of Emirati origin. Additionally, three patients were of Indian descent while six patients belonged to other ethnic groups. Among the 32 patients, seven (22%) presented with comorbidities, including eczema, arthropathy, delayed puberty, erythema nodosum, Glucose-6-phosphate dehydrogenase deficiency (G6PD) deficiency, primary sclerosing cholangitis, and Wegener's granulomatosis. Table 1 describes the study population characteristics. 

**Table 1:** Patient profile (Gender, Ethnicity, Comorbidities, and Age of Diagnosis) in the study cohort

	Gender	Ethnicity	Comorbidity	Age at diagnosis (in years)
1	Male	Arab (UAE)	None	13
2	Male	Arab (UAE)	None	11
3	Male	Arab (Syria)	None	14
4	Female	Arab (Algeria)	None	11
5	Female	Arab (Egypt)	None	9
6	Male	Arab (UAE)	None	10
7	Female	Pakistani	None	12
8	Male	Arab (UAE)	G6PD deficiency	11
9	Female	Arab (UAE)	None	4
10	Female	Hungarian	None	15
11	Male	Arab (Iraq)	None	15
12	Female	Arab (UAE)	None	7
13	Male	Serbian	None	0.16 (Two months)
14	Male	Indian	None	6
15	Male	Arab (UAE)	None	16
16	Female	Arab (UAE)	None	13
17	Female	Indian	Eczema	9
18	Male	Arab (UAE)	None	8
19	Female	Arab (Jordan)	None	11
20	Male	Arab (UAE)	Wegner granulomatosis	11
21	Male	Arab (UAE)	Primary sclerosing cholangitis	13
22	Female	Arab (Lebanon)	None	12
23	Male	Arab (Iraqi)	Arthropathy, delayed puberty	12
24	Female	Arab (Jordan)	None	14
25	Female	Indian	None	8
26	Female	Arab (UAE)	Arthropathy, delayed puberty, erythema nodosum	16
27	Male	British	Arthropathy , thalassemia trait	9
28	Male	Filipino	None	13
29	Female	Arab (Syria)	None	14
30	Male	Iranian	None	5
31	Male	Arab (UAE)	None	14
32	Male	Arab (UAE)	None	6

Out of the total, 84% of the cohort utilized the CDED, while 16% used EEN. Prior to the initiation of the diet therapy, the cohort reported experiencing gastrointestinal symptoms for a range of durations. Specifically, 70% of patients reported experiencing symptoms for less than eight months, with 50% reporting symptom duration of less than four months, and only 25% reporting symptom duration of less than one month. The remaining seven cases reported uncertain symptom duration.

The disease activity of the patients showed a variety of symptoms. Among the 32 patients, 10 (31.25%) had no abdominal pain, while six (18.75%), 12 (37.5%), and four (12.5%) reported mild, moderate, and severe abdominal pain, respectively. Almost all patients (31 out of 32) reported night symptoms, and approximately half (50%) reported weight loss. Abdominal tenderness was present in 20 patients. Additionally, 24 patients (75%) reported peri-anal disease (Table 2). The use of steroids sparing agents in the treatment of the patients was varied. Among the drugs used, azathioprine was the most common, with a usage rate of 40.5%. Adalimumab and infliximab were also frequently used, with a usage rate of 37.5% each. Mesalazine and methotrexate were used in 9% of the patients each. In a very small percentage of patients, ustekinumab and vedolizumab were used, with each drug being used in only one patient, approximately 3% of the total.

**Table 2:** Disease symptomatology and activity in the study cohort

	Disease activity							
	Abdominal pain	Defecation frequency (in 24 hours)	Night symptoms	Decrease in weight	Increase in height	Abdominal tenderness	Peri-anal disease	Extra-intestinal disease
1	Severe	2	No	No	No	Yes	No	No
2	Mild	Constipated	No	No	No	Yes	No	No
3	Mild to moderate	Unknown	No	Yes	No	No	No	Mouth ulcers
4	Mild to moderate	Unknown	No	Yes	No	Yes	No	No
5	Moderate to severe	Unknown	No	No	No	Yes	No	No
6	Nil	Unknown	No	No	No	No	No	Delayed puberty
7	Nil	Unknown	No	Yes	No	No	No	No
8	Moderate	4	No	Yes	No	Yes	Fissure	No
9	Nil	4	No	No	No	No	No	No
10	Yes	Unknown	No	Yes	No	No	No	No
11	Severe	Unknown	No	Yes	No	No	Fissure	No
12	Mild to moderate	Constipated	No	No	No	No	Fissure	No
13	Mild	10	No	No	No	No	Rectal prolapse and polyp	No
14	Nil	6	No	No	No	No	No	Mouth ulcers
15	Mild to moderate	Unknown	No	No	No	No	No	Mouth ulcers
16	Nil	2	No	Yes	No	No	No	Intrabdominal abscess
17	Nil	10	No	No	No	Yes	No	Mouth ulcer
18	Nil	12-	No	No	No	No	No	No
19	Mild to moderate	3	Yes	Yes	No	Yes	No	No
20	Nil	2	No	No	No	No	No	No
21	Mild to moderate	Unknown	No	Yes	No	No	No	No
22	Mild to moderate	3	No	Yes	No	Yes	Fissure and skin tag	No
23	Mild to moderate	2	No	No	No	No	No	Joint pain
24	Moderate	3	No	Yes	No	Yes	Fissure and skin tag	No
25	Nil	1	No	No	No	No	No	No
26	Mild	2	No	Yes	No	No	Skin tags	Joint pain and rash and erythema nodosum
27	Mild	1	No	Yes	No	No	No	Joint pain
28	Nil	Unknown	No	No	No	No	Fistula	No
29	Moderate to severe	2	No	Yes	No	Yes	No	No
30	Mild	5	No	No	No	No	No	No
31	Mild to moderate	2	No	No	No	Yes	No	No
32	Mild to moderate	5	No	Yes	No	Yes	No	Joint pain

**Figure 1:**
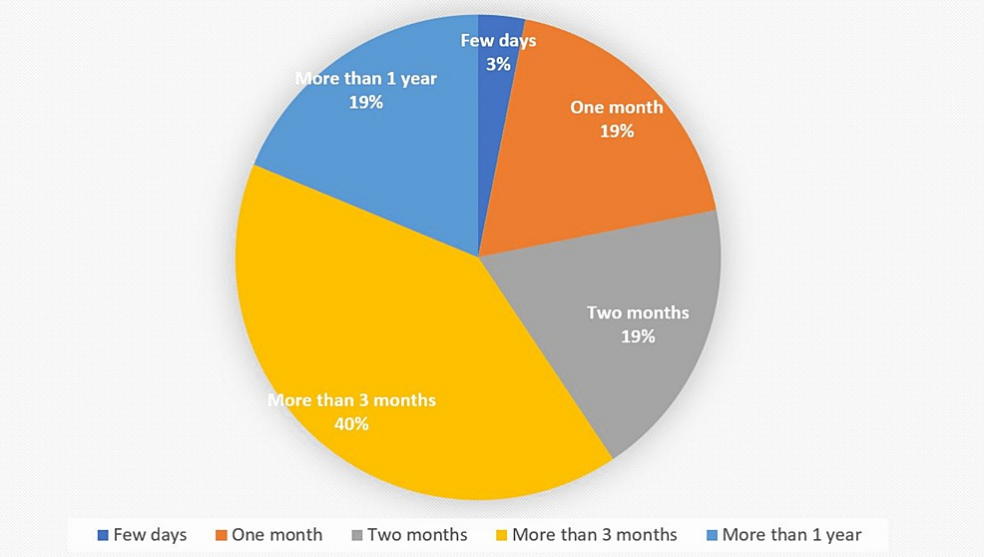
Duration of CDED/EEN utilization reported by the caregiver CDED: Crohn’s disease exclusion diet; EEN: Exclusion enteral nutrition

The data was able to identify the duration of the patient’s adherence to the prescribed diet plan. A small percentage of patients (3%) could only follow the diet plan for a few days (more than one week). A similar percentage of patients (19%) were able to follow the plan for one and two months, respectively. A larger percentage of patients (40%) were able to follow the diet plan for more than three months, which was considered a successful completion of phases one and two of the program. Finally, (19%) of patients were able to continue the diet plan for more than one year (Figure 1). According to the survey responses from parents, 56% reported strict adherence to the prescribed diet plan for their child, while (46%) did not follow the diet plan precisely. In regard to the weight change after starting the special diet, the majority of parents (56%) reported an increase in their child's weight, while 19% noticed a decrease in weight. A quarter of parents (25%) reported no change in their child's weight.

**Figure 2:**
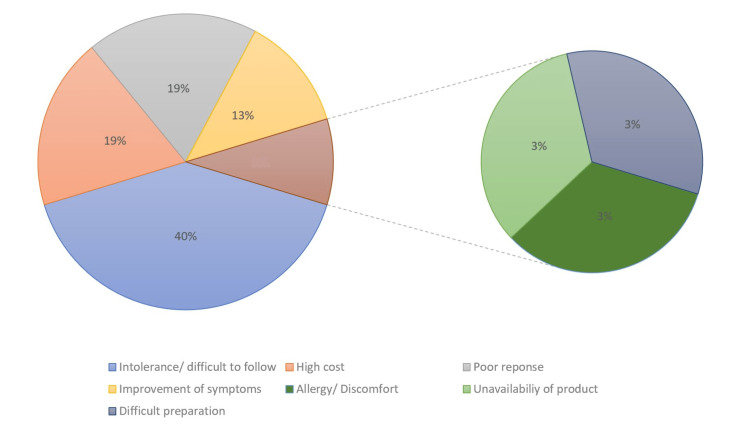
Causes of failure of dietary plan reported by the patient and the family

According to self-reported reasons for not completing the prescribed diet plan, 13 out of 32 patients (40%) found the plan very difficult to follow. Six patients (19%) considered the cost of the diet plan to be very high, and six patients (19%) discontinued the diet plan due to poor clinical response (persistence of symptoms). Four patients (13%) stopped the diet plan because their symptoms improved, one patient (3%) reported an allergy to a component of the diet plan, one patient (3%) experienced issues with the availability of the prescribed products, and one patient (3%) faced difficulties in preparing the diet plan (Figure 2).

## Discussion

In light of the ongoing increase in the number of patients with IBD and the complex nature of their cases, there is a pressing need to explore more effective and safer treatment modalities for this population. Our study has revealed a wide range of medications administered, which may indicate a lack of consistency in the response to initial therapy plans. This necessitated the escalation or switch of medications in an attempt to induce remission. Furthermore, in some cases, medications were replaced due to the occurrence of serious side effects, such as allergic reactions. These findings underscore the need for improved treatment strategies to address the complex nature of IBD while minimizing the risk of adverse events.

In our center, dietary therapy has emerged as a well-established and widely offered approach for managing IBD patients, both for induction and maintenance of remission. With its exceptional safety profile and high efficacy, this treatment modality demonstrates a promising future. The impact of diet on intestinal function and structure cannot be overstated [9]. Notably, a low-fiber diet has been linked to a reduction in mucus production and subsequent mucosal damage, leading to increased exposure to irritants, as evidenced by rat models [9]. Moreover, changes in food sources from plant to animal can also affect the gut's bacterial composition [9]. These observations emphasize the role of diet as a critical component in the management of IBD and call for further research to better understand the relationship between diet and IBD.

Diet management in Crohn’s disease

In our clinical practice, we adopt a cautious approach when selecting appropriate therapeutic options for patients with IBD, in collaboration with their paediatric gastroenterologist, while taking into consideration the disease severity and social circumstances. Two primary types of dietary therapy that we employ include EEN and CDED. EEN involves the complete elimination of food intake for a period of 6-8 weeks, with caloric requirements fulfilled via specialized liquid enteral formulas named "Modulen® IBD." This approach is primarily utilized for the induction of remission and is typically recommended for short-term use due to its challenging nature.

Subsequent to the EEN phase, patients are typically transitioned to Phase 1 of the CDED. In some cases, patients may bypass the EEN phase entirely and begin directly with Phase 1 of CDED. During this phase, 50% of the patient's energy and nutrient intake are derived from Modulen® IBD, while the remaining 50% are sourced from five mandatory foods, namely chicken breast, eggs, potatoes (fresh, cooked, and peeled), bananas, and apples. The patient is allowed to replace chicken with fresh lean fish. Other food items are also allowed and usually discussed with the patient and the parents in detail during their visit to the dietitian. It is crucial to closely monitor patients for signs and symptoms of intolerance, such as diarrhea, vomiting, and other gastrointestinal disturbances, particularly upon the initial introduction of the formula. It is recommended that patients and caregivers are advised accordingly.

After the completion of Phase 1, patients proceed to Phase 2 of the CDED after six weeks. In this phase, the Modulen® IBD formula portion is reduced to 25%, while the mandatory foods remain a part of the diet. Additionally, patients are permitted to consume some additional items (more than phase 1), such as limited quantities of fish, select legumes, carbohydrates (white rice), and fruits. This phase marks a step towards a more balanced and diversified diet. Following completion of both Phase 1 and Phase 2 of the CDED, patients progress to Phase 3, typically after a total of 12 weeks. During this phase, patients begin incorporating additional foods gradually, with the goal of transitioning back to a regular, healthy diet.

EEN versus CDED

In comparison to EEN, CDED has demonstrated higher tolerability at the six-week mark, with statistically significant findings and improved compliance rates. Both approaches have exhibited nearly identical response rates and remission rates. After six weeks of CDED implementation, a substantial decrease in paediatric Crohn's disease activity index (PCDAI) was observed, equivalent to that of EEN, accompanied by a significant decline in C-reactive protein (CRP) levels. Furthermore, after 12 weeks, CDED was able to maintain low CRP and PCDAI levels, equal to or below 10, whereas EEN demonstrated a lesser ability to do so. This may be due to the challenges that patients encounter in adhering to the rigorous EEN plan [10]. Rotem et al. have demonstrated 82% effectivity of CDED to induce remission and drop of CRP to normal level at 3 weeks of treatment [7]. Overall, Levine et al. have shown that CDED can be tolerated by the patient 13 times more than EEN [10].

The patient and family perspective on the CDED plan

Through our survey, we have identified several underlying factors that hinder patients' adherence to the CDED plan. The majority of patients reported difficulties in strictly following the plan, particularly in adhering to the strict dietary restrictions and details. The early phases of the CDED, which require patients to consume formula and limit their food choices, presented a significant challenge. Additionally, children with CD might be picky eaters with selective food preferences, which posed a significant barrier to their compliance with the CDED.

Moreover, our survey showed that some patients discontinued the CDED due to misconceptions that the diet had failed or had successfully induced remission, and therefore was no longer necessary. To mitigate these misconceptions, treating physicians and dietitians must emphasize the importance of continuing the diet even after clinical recovery. Patients need to understand that in certain cases, the disease may be aggressive and resistant, and the CDED needs to be maintained even if the response to treatment is not optimal. Studies have demonstrated that adding CDED can be effective in cases where biological therapy has failed [11].

Surprisingly, the cost of the formula or dietary items was not reported as a significant factor in discontinuing the CDED by families. This finding is contrary to our initial assumptions that the cost may impact patients' ability to adhere to the diet plan, particularly since the formula is not covered by most health insurance companies in the UAE.

To our knowledge, there is a lack of literature discussing the causes of poor compliance rates to dietary plans from the patient's perspective. Our survey provides valuable insight into the actual reasons for patients' failure to complete dietary plans, highlighting the need to address these factors to improve compliance rates which is essential for inducing remission and healing.

Dietitian support in CDED

Adequate patient education and ongoing support from a trained paediatric dietitian are crucial for the success of these dietary interventions. However, we have observed that many CD patients only attend a single consultation with a dietitian and do not follow up thereafter. Additionally, 47 % of our patients did not attend dietitian clinics at all, opting instead to collect the special formula only. This lack of follow-up and support may indirectly contribute to difficulty adhering to the prescribed diet plan, which can significantly impact patient outcomes.

Unfortunately, the cost of ongoing dietitian support is often a significant barrier for patients and their families, as health insurance companies typically do not cover this expense. This financial burden can further reduce the likelihood of patients following the prescribed dietary plan, which in turn may negatively impact remission rates. Ultimately, patients who are unable to follow the dietary plan may require recurrent hospital visits and admissions, which are more costly than regular clinic visits to a dietitian. To help the patient to adhere to their dietary plan, Clinicians can complete the CDED training modules available at www.modulifexpert.com which is a good resource for the patient and the family as well. Clinicians can enhance their knowledge and skills, while patients can utilize the website and mobile app for additional support and resources throughout their treatment journey. Lastly, addressing these challenges and finding solutions to increase patient access to ongoing dietitian support may have a significant impact on the management of CD and improve patient outcomes.

This study has limitations that warrant consideration when interpreting its findings. The sample size is relatively small, and data collection relied on patient and family-completed questionnaires, potentially introducing recall bias. Furthermore, essential socio-economic variables such as family education and financial background were not accounted for in the analysis.

## Conclusions

In conclusion, managing inflammatory bowel disease, and specifically Crohn’s disease, requires a multi- modular and a multidisciplinary approach . Diet role has been highlighted as an important modality in inducing remission and maintaining a healthy gut. Despite the proven benefits, adherence to specialized diets such as the Crohn’s disease exclusion diet can be challenging for patients and we had identified several obstacles based on the patient’s opinion. As healthcare providers, we must address the barriers to adherence and prioritize the role of dietitians in managing inflammatory bowel disease patients, providing necessary education and support, and ensuring the successful integration of dietitian service for improved patient outcomes.

## Appendices

Dietary management of Crohn's disease in paediatrics: United Arab Emirates (UAE) experience questionnaire

Personal Information ( To be filled in by the data collector)

Gender: Male, Female 

Age : 

Ethnicity:

Disease course information (to be filled in by the data collector)

1. For how long have the symptoms been presented?

o    Less than 1 month

o    2 months

o    3 months

o    4 months

o    5 months

o    6months

o    7 months

o    8 months

o    9 months

o    9-12 months

o    More than 1 year

o    Uncertain

2. Symptoms

o   Abdominal pain (yes, no)

o   Defection frequency in 24 hours :

o   Night symptoms : (yes, no)

o   Wight Decrease : (yes, no)

o   Height Increase : (yes, no)

3. How severe were the symptoms on presentation?

o    Mild

o    Moderate

o    Severe

4. For how long was the treatment used before starting diet management?

o   More than 3 months

o   More than 6 months

o   More than 1 year

5. Any other comorbidities with IBD?

o   Yes, enumerate

o   No

6. If yes, what are the comorbidities?

o   Skin condition

o   Lung disease

o   Joint disease

o   Endocrine disease

o   Other (             )

7. if yes, what is the name of the medication used for the comorbidities if any?

8.    Any Family history of IBD?

o   Yes

o    No

Diet management information (to be filled in by the participant)

1. What was the diet plan? Explain (Either EEN or CDED) 

2. Any medication used with the diet?

o   Yes

o   No

3. If yes, what type of medications are used with the diet plan?

o   Anti-inflammatory (Ibuprofen) 

o   Steroids (Prednisolone) 

o   Immunosuppressant (Azathioprine/ Imuran, Methotrexate) 

o   Biologic (yes or no) 

4. If yes, what was initiand first, medication or the diet plan?

o   Medication

o   Diet therapy

o   Combination

5. If Diet was added to the medication; after how long of using the medication?

o   More than 3 weeks

o   More than 3 months

o   More 6 months

6. If medication was stopped and diet was initiated later; after how long of stopping the medication?

o   After 3 weeks

o   After 3 months

o   After 6months

7. For how long have you been on diet management?

o   Few days

o   One month

o   2 months

o   More than  3 months

o   More than a year

8. How frequently have you/your child had follow-up appointments with the gastroenterology doctor?

o   Every 2 weeks

o   Every 4 weeks

o   Every 6 weeks

9. How your/your child's weight has changed after starting diet therapy?

o   Increased

o   Decreased

o   Stable

10. Did you complete the diet plan or was it stopped?

o   Completed

o   Stopped

11.  If it was stopped by the parents/ patient, what was the reason? (can choose more than 1 answer)

o   High Cost

o   Intolerance/ Difficulty to follow

o   Poor response

o   Improvement of symptoms

o   Allergy/Discomfort

o   Unavailability of product

o   Difficult preparation

Other ………….

## References

[1] McDowell C, Farooq U, Haseeb M (2023). Inflammatory Bowel Disease. Treasure Island (FL): StatPearls.

[2] Kirsner JB (2001). Historical origins of current IBD concepts. World J Gastroenterol.

[3] Kuenzig ME, Fung SG, Marderfeld L (2022). Twenty-first century trends in the global epidemiology of pediatric-onset inflammatory bowel disease: systematic review. Gastroenterology.

[4] Ye Y, Manne S, Bennett D (2018). Pediatric inflammatory bowel disease in the U.S.: population-based prevalence estimates from large national databases. Am J Gastroenterol.

[5] Narula N, Wong EC, Dehghan M (2021). Association of ultra-processed food intake with risk of inflammatory bowel disease: prospective cohort study. BMJ.

[6] Levine A, Rhodes JM, Lindsay JO (2020). Dietary guidance from the International Organization for the Study of Inflammatory Bowel Diseases. Clin Gastroenterol Hepatol.

[7] Sigall Boneh R, Van Limbergen J, Wine E (2021). Dietary therapies induce rapid response and remission in pediatric patients with active Crohn's disease. Clin Gastroenterol Hepatol.

[8] Bischoff SC, Bager P, Escher J (2023). ESPEN guideline on clinical nutrition in inflammatory bowel disease. Clin Nutr.

[9] Levine A, Sigall Boneh R, Wine E (2018). Evolving role of diet in the pathogenesis and treatment of inflammatory bowel diseases. Gut.

[10] Levine A, Wine E, Assa A (2019). Crohn's disease exclusion diet plus partial enteral nutrition induces sustained remission in a randomized controlled trial. Gastroenterology.

[11] Sigall Boneh R, Sarbagili Shabat C, Yanai H, Chermesh I, Ben Avraham S, Boaz M, Levine A (2017). Dietary therapy with the Crohn's disease exclusion diet is a successful strategy for induction of remission in children and adults failing biological therapy. J Crohns Colitis.

